# Associations between Mobile Internet Use and Self-Rated and Mental Health of the Chinese Population: Evidence from China Family Panel Studies 2020

**DOI:** 10.3390/bs12070221

**Published:** 2022-07-01

**Authors:** Haifeng Ding, Chengsu Zhang, Wan Xiong

**Affiliations:** 1 School of Public Administration and Law, Hunan Agricultural University, Changsha 410100, China; haifeng@stu.hunau.edu.cn; 2 School of Management, University of Sanya, Sanya 572000, China; 3 School of Business, Shanghai University of Finance and Economics, Shanghai 200434, China; xiongwan@163.sufe.edu.cn

**Keywords:** mobile Internet use, self-rated health, mental health, China, CFPS

## Abstract

With societal and technological developments, mobile Internet has become the most popular and widespread means to use the Internet in China. Thus, exploring the relationship between mobile Internet use and the self-rated health and mental health of the Chinese population is of great importance. This study empirically examined the impact of mobile Internet use on residents’ health using data from the China Family Panel Studies 2020 and conducted a heterogeneity analysis. The results revealed a significant negative association between mobile Internet use and the self-rated health of the population, but a significant positive association was found relative to their mental health. The results of this analysis passed a robustness test. The results of the heterogeneity analysis showed that mobile Internet use had a more significant association with the health of residents with secondary school education and university education compared to those with primary school education or below and graduate education. Furthermore, this study addresses the endogeneity problem using the propensity-score matching model, which is shown to be better at eliminating sample selectivity bias. If endogeneity is not addressed, the negative association with mobile Internet use on residents’ self-rated health will be underestimated and its positive association with their mental health will be overestimated. The Chinese government should issue guidelines on the duration of Internet use, strictly regulate exaggerated and harmful content on mobile network platforms, and strengthen people’s online skills through training to improve their digital literacy, especially for rural populations.

## 1. Introduction

Health is important for the development of populations and societies. Since the reform and opening up of China, there have been rapid developments in medical and health care in the country, and the health condition of the residents has hugely changed [1]. According to data from the Chinese Health Care Commission, by 2020, life expectancy in China will reach 77.93 years, an increase of nearly 43 years compared to a life expectancy of merely 35 years in the early years after the founding of the country; in this regard, China is already catching up with some developed countries [2]. In 2017, at the 19th Congress of the Communist Party of China (CPC), President Xi Jinping proposed the “Health China Strategy”, with an aim of improving the health of the population to achieve the goal of a healthy and strong country and, thereby, enhance the happiness of the population [3,4]. In 2021, the Sixth Plenary Session of the 19th CPC Central Committee further reiterated the need to strengthen the construction of a healthy China [5]. The Chinese government has always put the health of the people first. However, with developing times and society, intelligent digital technologies represented by the Internet have permeated every aspect of people’s daily lives, impacting them in profound ways [6]. According to the latest data from the China Bureau of Statistics, as of 2020, 989 million people had gained access to the Internet in China, including 986 million cell phone users, making China the country with the highest number of Internet users worldwide [7]. On the one hand, Internet technology has facilitated people’s daily lives; in particular, the use of mobile Internet via cell phones has brought great convenience to people [8]. On the other hand, the emergence of the Internet has had some negative effects on people, particularly in the form of addiction to the Internet [9]. The health ecology theory posits that the environment has a multi-level impact on individuals and the complexity of the influencing factors [10]. In this context, the current study aims to examine whether mobile Internet use affects the health of the population.

In recent years, scholars have explored the relationship between technological progress and population health. For example, using data from a survey in China, Yang and He found that Internet use had a significant positive impact on population health [11]. Similarly, Zhao and Liu analyzed the impact of Internet use on older adults using 2015 CGSS data and found that Internet use significantly contributed to the physical and mental health of older adults [12]. Li and Ding [13], Yang and Gu [14], and Xu and Lai [15] also reported the positive impact of Internet use on the population’s health. However, other studies have reported contrasting results. Niu et al. found that social networking site use had a significant negative predictive effect on depression in older adults [16]. In the U.S., Matusitz found that Internet use had a negative impact on the health of the residents, specifically because Internet use can lead to sedentary, inactive behavior, which in turn puts people at increased risk of weight gain and its related complications [17]. Furthermore, Hökby pointed out that the frequency of Internet use and access to Internet use all had a negative impact on adolescent health [18]. Choi’s survey of 1248 adolescents in Korea found that excessive Internet use can have a negative impact on adolescent health [19]. Similarly, Ning et al. found that excessive Internet use can have a negative impact on the health of rural adolescents [20].

Thus, the above studies indicate that the extant literature has not yet reached a consensus on the connection between the Internet and people’s health. It is worth noting that some studies have focused on the Internet as a single entity while not differentiating general Internet use from the use of mobile Internet. However, the majority of Internet users in China use mobile devices to access the Internet. Thus, this study explores the relationship between mobile Internet use and health using data from the latest China Family Panel Studies 2020 (CFPS), with the aim of providing new empirical evidence to further understand technological advances and their impact on population health.

## 2. Materials and Methods

### 2.1. Datq Sources

This study uses data from the latest China Family Panel Studies 2020 (CFPS). The CFPS is carried out by the Institute of Social Science Survey Center of Peking University, China, with a large sample size and strong representation. CFPS was launched in 2010. The survey covered 25 provinces (municipalities and autonomous regions) across the country. The CFPS sample is a multi-stage probability sample drawn using the implicit stratification method. The multi-stage sampling design was adopted both to reduce the operational costs of the survey and to take into account the social contextual differences in Chinese society. In terms of sampling design, the CFPS database first divides the 25 provinces/municipalities/autonomous regions in China into two categories: One category of provinces and municipalities is the inferred sample at the provincial level to meet the requirements of provincial inference. Five provinces/municipalities were selected for these data, namely, Liaoning, Shanghai, Henan, Guangdong, and Gansu, also known as large sample provinces. The second category of provinces/municipalities include the 20 provinces/municipalities/autonomous regions outside the above 5 provinces/municipalities. The sample size of this category of provinces/municipalities does not allow for inference at the provincial level and is also referred to as small sample provinces. The weighing of the data from these two types of samples provides a valid estimate of the overall 25 provinces/municipalities/autonomous regions, which can then be used to make inferences for the entirety of China. The survey involved 16,000 households, and it was conducted once every 2 years. Only adult data were published for the 2020 CFPS, with a total of 28,590 adult respondents. The 2020 questionnaire includes modules on basic information, educational status, income, and health care. The sample was screened, and responses with missing data and outliers were eliminated, resulting in a valid sample size of 7962. As CFPS is a national questionnaire, it involves various types of respondents from different populations, different occupational backgrounds, and different regions. Therefore, when faced with the limitations of different research questions and various types of variables, there may be too much data missing. This is still generally representative of the Chinese population.

### 2.2. Design

The independent variable in this study is mobile Internet use and the dependent variable is the self-rated health and mental health of the residents. According to previous studies [21,22,23], self-rated health is a reasonable indicator of the population’s comprehensive judgment of their own health over a long period of time, and the validity of this indicator has been confirmed by several international and national studies. To better reflect the level of health, in this study, self-rated and mental health were selected as a comprehensive measure of health status. In the 2020 CFPS questionnaire, self-rated health is measured with the question, “How healthy do you consider yourself to be?” The responses are rated on a 5-point scale with the following options: “very healthy = 1”, “healthy = 2”, “relatively healthy = 3”, “fair = 4”, and “unhealthy = 5”. Psychological health is measured with the question, “How often did you feel sad and upset in the past week?” The responses include “hardly ever = 1”, “sometimes = 2”, “often = 3”, and “most times = 4”. To assess the explanatory variable, mobile Internet use, the questionnaire includes the question, “Do you use mobile devices to access the Internet, such as cell phones, tablets, etc.?” The response is taken on a dichotomous scale of “no = 0” or “yes = 1”. Additionally, as population health is believed to be affected by other factors, personal characteristics, such as gender and age, and lifestyle variables, such as the presence of smoking and drinking are included as control variables. The definitions of the variables and the results of the descriptive statistical analysis are shown in Table 1.

### 2.3. Model Design

Since the dependent variable in this paper is a multicategorical variable, an Ordered Probit regression model is set to analyze the relationship between mobile Internet use and the physical and mental health of the population [24,25]. The specific model settings are as follows.

(1) $${\mathrm{Health}}_{i}{=\alpha +\beta \mathrm{Mobile}\;\mathrm{Internet}}_{i}+\gamma I{+\varepsilon }_{i}$$

In Equation (1), ${\mathrm{Health}}_{i}$ is the health level of the population, ${\mathrm{Mobile}\;\mathrm{Internet}}_{i}$ indicates the use of mobile Internet by the population, $D_{i}$ is the control variable that has an effect on the health status of the population, and $\varepsilon_{i}$ is a random disturbance term. Limited by differences in endowment resources, significant heterogeneity can occur in the impact of Internet use on the health of different populations. To further explore the association between mobile Internet use and population health, we examined heterogeneity from a literacy perspective. Similarly, we use the Oprobit regression model for estimation. The model is shown in Equation (1). Limited by endowment differences, different populations’ Internet use behavior is an independent choice based on their own judgment. Therefore, in order to reduce bias, this paper uses a propensity score matching (PSM) model to estimate the net effect of mobile Internet use on population health effects [26,27]. All analyses were conducted using STATA (version 15.0, StataCorp., College Station, TX, USA).

## 3. Results

### 3.1. Baseline Regression Analysis

As seen in Table 2, there is a significant connection between mobile Internet use and the self-rated health and mental health of the Chinese population. There is a negative correlation between mobile Internet use and self-rated health. However, there is a positive correlation between mobile Internet use and the mental health of the population. Models (a) and (c) include only personal characteristic variables. The results reveal that those using mobile Internet showed a 0.126 unit change in self-rated health in a negative direction and a 0.117 unit change in mental health in a positive direction compared to those not using mobile Internet, and they were significant at 1% and 5% significance levels, respectively. Models (b) and (d), which incorporate all the control variables, show a change of 0.125 units in a negative direction for self-rated health and a 0.118 unit change in a positive direction for mental health in those using mobile Internet compared to those not using it, and they are significant at the 1% and 5% levels of significance, respectively. The above results indicate that mobile Internet use has a significant negative connection with the self-rated health of the population and a significant positive connection with the population’s mental health. 

Regarding the control variables, in terms of gender, men showed better self-reported health status than women. In terms of age, self-rated health deteriorated with increasing age, whereas mental health improved. In terms of marital status, marital status is negatively associated with the mental health of the population; however, there was no significant association with marital status on self-rated health. In terms of literacy, higher levels of education were associated with poorer self-rated health but better mental health. In terms of lifestyle, smoking was related to poorer health status. Finally, health status increased with increasing frequency of exercise, indicating that physical activity is an important way to improve health.

### 3.2. Robustness Tests

In order to ensure the robustness of the model results, robustness checks were performed using a substitution econometric model approach. Since the dependent variable in this study is a multicategory variable, the ordered logit model was used as a replacement model for the analysis. The results of the robustness tests are shown in Table 3. As seen in Table 3, Models (e) and (g) included only individual characteristic variables, and Models (f) and (h) included all the control variables; both were significant. In terms of self-rated health, those using mobile Internet were less healthy than those not using mobile Internet, indicating that the use of mobile Internet is associated with significantly lower population health. In contrast, the mental health of those using mobile Internet was better than that of non-users, indicating that mobile Internet use is associated with better mental health in the Chinese population. This result is consistent with the results of the benchmark regression, indicating that the results of the empirical analysis in this study are robust.

### 3.3. Heterogeneity Analysis

Limited by differences in endowment resources, significant heterogeneity can occur in the impact of Internet use on the health of different populations. To further explore the association between mobile Internet use and population health, we examined heterogeneity from a literacy perspective. The results of the heterogeneity analysis are shown in Table 4. The impact of mobile Internet use on population health differed significantly by the level of education. The highest level of significance was found in the population with secondary school education, and it was significant at the 1% level, followed by those with university education, and these were significant at the 10% level of significance. This suggests that mobile Internet use significantly reduces the self-rated health of people with secondary and tertiary education, while it does not have a significant association with people with primary and lower education and postgraduate education. In terms of mental health, mobile Internet use was significantly related to better mental health among the secondary school-educated group, but no significant effect in the other groups was observed.

### 3.4. Endogenous Elimination

Individual mobile Internet use is influenced by a series of factors, including age, income, and education level; it is an individual choice made by individuals based on their own endowment resources. Therefore, the model can generate endogeneity problems. To overcome this problem, this study used a propensity score matching model. Compared to baseline regression, the PSM model can effectively eliminate the effect of confounding variables on the estimation results [28,29]. As a result, the relationship between mobile Internet use and the self-rated health and mental health of the population can be described more accurately. Drawing on previous studies, four methods, K-nearest neighbor matching, K-nearest neighbor caliper matching, radius neighbor matching, and kernel matching, were used for estimation in this study. Additionally, to ensure excellent matching results, a balance test was performed on the sample phi quality. A significant difference between the two groups of samples after matching indicates poor matching and vice versa. The results of the sample matching quality balance test are shown in Table 5.

**Figure 1:**
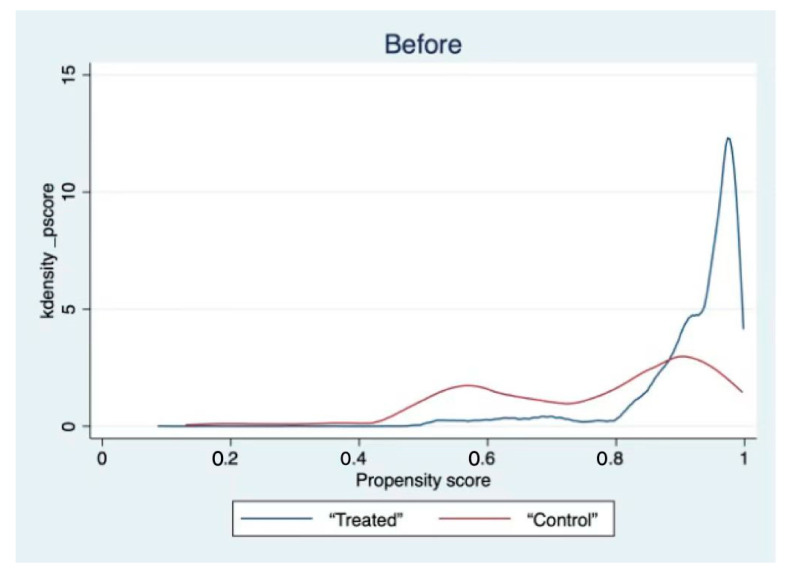
Kernel density function plot (before matching).

**Figure 2:**
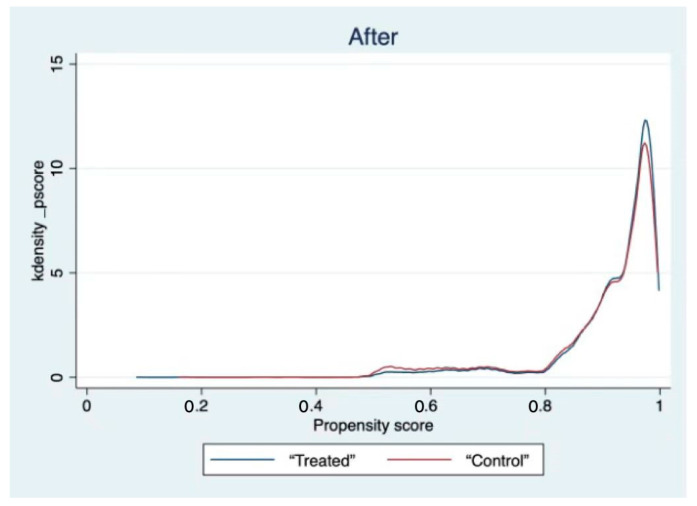
Kernel density function plot (after matching).

Table 5 shows that all the variables are well matched. Additionally, kernel density function plots are reported (see Figure 1 and Figure 2). The graph shows that the matched curves of the treatment and control groups overlap, indicating that the treatment and control groups are well matched, which effectively eliminates the possibility of sample selectivity bias.

Table 6 shows that, using the K-nearest neighbor matching method, the values of the average treatment effect on the treated for the effects of mobile Internet use on the self-rated and mental health of the population were 0.057 and −0.087 before matching and 0.183 and −0.063 after matching, respectively. After controlling for sample selectivity bias, the net effects of mobile Internet use on the self-rated health and mental health of the population were 18.3% and 6.3%, respectively. Other matching methods showed similar results. The net effects of mobile Internet use on population health calculated via K-nearest neighbor caliper matching, radius neighbor matching, and kernel matching were 19.4%, 19.9%, and 22.7% and 7.2%, 6.7%, and 7.8%, respectively, for self-rated and mental health, respectively. The results obtained with propensity score matching is robust. Moreover, the results show that if endogeneity is addressed, and we will underestimate the negative association between mobile Internet and population health, whereas the contribution of mobile Internet use to the mental health of the population would be overestimated.

## 4. Discussion

### 4.1. Summary of Findings

According to the empirical results, mobile Internet use has a significant negative association with the self-rated health of the population. This result is inconsistent with the results of previous related studies [11,12,13,30]. The possible reasons for this may be, on the one hand, related to the selection of the data sample and the subjects of the study, and on the other hand, may be closely linked to the current state of development and the Internet environment in China. Currently, China’s Internet penetration rate has reached 73%, and the number of Internet users has reached 1.032 billion, bringing China to the first rank worldwide in terms of the number of Internet users [31]. While this tremendous growth in the number of users shows the rapid development of China’s network infrastructure, it also calls for caution against the potential adverse effects of mobile Internet use. The Internet is a product of technological advances that have enhanced efficiency and made people’s lives convenient, but attention should also be paid to its negative effects [32]. First, mobile Internet affects the self-rated health of the population by reducing available time for daily socialization and exercise. Observation indicates that, with access to mobile Internet, particularly the currently highly popular short video platforms (APP), people tend to spend much of their time browsing the Internet, which greatly eats into the time that should be spent on communicating with family and friends or engaging in physical exercise, thus greatly affecting people’s health [31,33]. Second, to attract more traffic, some mobile Internet platforms create a lot of “fascinating” information, and such “dazzling” information affects people’s health [34]. This quality of Internet content makes people with low self-control ability, especially teenagers, become addicted to it and they are unable to extricate themselves, which has a significant association with population health [35,36,37]. Contrary to the findings of self-rated health, mobile Internet use was seen to contribute significantly to the mental health of the population, which is inconsistent with the findings of some existing studies, such as those by Xie et al., Pantic, and Chambers et al. [38,39,40]. This may be due to the rapid development of the Internet in China; Internet technology has changed from rapid development to high-quality development; instead of being satisfied with low level material enjoyment, users are more interested in the Internet’s spiritual impact in their lives [41,42]. In particular, in the modern environment with high pressures of life and work, mobile Internet allows people to read, play, and socialize anytime and anywhere, breaking through many limitations of time and space [43,44,45]. This greatly enriches people’s mental lives, thus enhancing their mental health.

The results indicate significant heterogeneity in the effect of mobile Internet use on the health of people with different levels of education. The connection of mobile Internet on the health of people with elementary school education and below was not significant. This result is similar to the results of Wang’s analysis [46]. This may be because this group is often excluded from the Internet wave due to their lower education. This indicates that there is a clear “digital divide” and, therefore, the digital literacy of this group should be improved in the future [47,48,49]. Similarly, the results show that the connection of mobile Internet use on the health of people with graduate education is insignificant, but the contribution effect is positive. Highly educated people have a strong knowledge reserve and better self-control abilities. When faced with complex information on the Internet, they can effectively screen it; thus, the impact on their health is not obvious. In addition, highly educated people are better equipped to use Internet resources to improve their health by using various means. Among all users, mobile Internet use has the most significant association with the health of people with secondary school education. This population tends to have basic Internet skills but not a very rich knowledge base. Therefore, they are most vulnerable to the impact of the Internet. When confronted with fascinating information, they can become addicted to it, which can eventually affect their health. In terms of mental health, mobile Internet use had a significant association with health improvement for people with secondary education, but not for other groups. The possible reason for this is that the Internet is the main entertainment channel for this group of people; thus, Internet use may improve their mental health.

### 4.2. Policy Implications

First, the government should issue guidelines on the duration of Internet use and guide citizens to use the Internet in moderation. According to relevant data, China’s Internet users spend approximately 28.5 h per week online, with an average of more than 4 h per day in the morning. Therefore, relevant policies should be formulated in the future to guide the population to access the Internet in moderation. For example, by setting reminders in mobile apps or cell phones, when the screen use time exceeds a certain limit, the phone will automatically sound an alarm to guide people to control their time of Internet use and avoid Internet addiction and dependence. Second, it is advised that the content created and encouraged by mobile network platforms should be strictly supervised to create a healthy network environment. To tap into a larger “viewership”, some online platforms create exaggerated content to attract people’s attention, which leads to people with poor self-control falling prey to it and become unable to extricate themselves. In particular, younger people, whose world views and values have not yet matured, are easily influenced by harmful and exaggerated information. Therefore, the supervision of online platforms should be increased in the future to strictly control the dissemination of undesirable information and block such information from the source to prevent its harmful association with the self-rated health and mental health of the population. Third, the population’s Internet skills should be strengthened and their Internet literacy should be improved. Most Internet users in China reside in rural areas. Limited by literacy and material conditions, they tend to have low digital literacy and are easily influenced by the Internet. Therefore, the government should strengthen the Internet skills of users in rural areas, guide them to access the Internet in a healthy manner, and improve their digital literacy through training and other means. For example, community-based training can be conducted from time to time to spread general knowledge of the Internet among the population and improve their digital literacy level and information screening ability.

### 4.3. Innovations and Limitations

The innovations of this study are as follows. First, this study empirically examines the relationship between mobile Internet use and the self-rated health and mental health of the Chinese population from the perspective of mobile Internet, using the most recent nationally representative data. This provides new empirical evidence to understand the relationship between Internet use and health over time. Second, this study takes into account the sample self-selection problem. The propensity score matching model is used to eliminate the sample’s selectivity bias, thus ensuring the robustness of the model estimation results. This study also has some limitations. First, the health status of the population is a long-term dynamic development process; thus, using cross-sectional data will inevitably lead to biased results. Therefore, we will further test this relationship in the future using multi-year tracking data. Second, this study did not examine how different mobile Internet apps and Internet usage time affect the self-rated health and mental health of the population. This question will be the focus of studies in future research. Third, in terms of indicator selection, the measurement questions selected in this paper regarding mobile Internet use are too general. This may have some measurement error. In addition, in terms of mental health, we used “feel sad and upset” as a measure, which may not be representative of the overall mental health of the population. Therefore, these areas are where we will make further breakthroughs in the future.

## 5. Conclusions

This study empirically examines the relationship between mobile Internet use and the self-rated and mental health of the Chinese population using data from the 2020 China Family Panel Studies. The results showed a significant association between mobile Internet use and self-rated and mental health. In terms of self-rated health, there was a negative relationship between mobile Internet use and self-rated health. In terms of mental health, there was a positive relationship between mobile Internet use and mental health. The degree of negative impact of mobile Internet use on self-rated health is higher than the degree of positive impact on mental health. Heterogeneity analysis showed significant heterogeneity in the effects of mobile Internet use on the health of people with different levels of education. The association of mobile Internet use on the health of people with secondary school and university education was more significant than that on people with elementary school education and below and graduate education. We also performed robustness tests using a substitution analysis model. The results show that the conclusions obtained from this study are well robust. In addition, we used a propensity score matching model to estimate the net effect of mobile Internet use on the demographic impact. The results show that PSM model is effective in eliminating sample-based bias. If endogenous problems are not eliminated, the negative relationship of mobile Internet use on the self-rated health of the population will be underestimated, whereas its contribution to mental health will be overestimated.

## Figures and Tables

**Table 1:** Variable assignment and descriptive statistics.

Variable	Definition	N	Mean	SE
Dependent variable				
Self-rated health	Very healthy = 1, healthy = 2, relatively healthy = 3, Fair = 4, Unhealthy = 5	7962	2.587	1.023
Mental Health	Almost never = 1, sometimes = 2, often = 3, most of the time = 4	7962	1.567	0.642
Independent variable				
Mobile Internet Usage	No = 0, Yes = 1	7962	0.900	0.300
Control variable				
Gender	Female = 1, Male = 2	7962	0.334	0.472
Age	Unit: years	7962	30.513	8.325
Marital Status	Unmarried = 1, in marriage = 2, divorced = 3	7962	-	-
Education level	Primary and below = 1, Secondary = 2, University = 3, Graduate = 4	7962	-	-
Medical Insurance	No = 0, Yes = 1	7962	0.868	0.339
Smoking status	No = 0, Yes = 1	7962	0.069	0.253
Drinking situation	No = 0, Yes = 1	7962	0.046	0.209
Frequency of physical exercise	Very little/almost no = 1, less = 2, average = 3, more = 4, many = 5	7962	1.661	1.065

**Table 2:** Baseline regression results.

Variable	Model (a)	Model (b)	Model (c)	Model (d)
	Self-Rated Health	Self-Rated Health	Mental Health	Mental Health
Mobile Internet Usage	0.126 *** (0.043)	0.125 *** (0.043)	−0.117 ** (0.046)	−0.118 ** (0.046)
Gender	−0.131 *** (0.026)	−0.152 *** (0.028)	−0.277 *** (0.029)	−0.293 *** (0.031)
Age	0.032 *** (0.002)	0.032 *** (0.002)	−0.007 *** (0.002)	−0.007 *** (0.002)
Marital Status	0.004 (0.026)	−0.003 (0.027)	0.099 *** (0.028)	0.097 *** (0.029)
Education level	0.045 *** (0.019)	0.048 ** (0.019)	−0.043 ** (0.021)	−0.040 * (0.021)
Medical Insurance		0.018 (0.036)		−0.041 (0.039)
Smoking status		0.164 *** (0.051)		0.098 * (0.056)
Drinking situation		−0.062 (0.060)		−0.003 (0.066)
Frequency of physical exercise		−0.022 * (0.012)		−0.009 (0.013)
N	7962	7962	7962	7962
Adj-X^2^	0.0236	0.0243	0.0094	0.0098

Note: *, **, *** indicate significance at the 10%, 5%, and 1% levels, respectively.

**Table 3:** Robustness test results.

Variable	Models (e)	Models (f)	Models (g)	Models (h)
	Self-Rated Health	Self-Rated Health	Mental Health	Mental Health
Mobile Internet Usage	0.243 *** (0.078)	0.243 *** (0.078)	−0.139 * (0.080)	−0.141 * (0.080)
Gender	−0.243 *** (0.045)	−0.278 *** (0.049)	−0.495 *** (0.048)	−0.521 *** (0.053)
Age	0.057 *** (0.003)	0.056 *** (0.003)	−0.013 *** (0.003)	−0.013 *** (0.003)
Marital Status	0.007 (0.046)	−0.006 (0.046)	0.173 *** (0.049)	0.169 *** (0.049)
Education level	0.083 *** (0.033)	0.088 *** (0.034)	−0.060 * (0.035)	−0.056 (0.035)
Medical Insurance		0.037 (0.063)		−0.037 (0.067)
Smoking status		0.299 *** (0.090)		0.163 * (0.095)
Drinking situation		−0.119 (0.108)		−0.023 (0.112)
Frequency of physical exercise		−0.039 * (0.020)		−0.012 (0.021)
N	7968	7968	7962	7962
Adj-X^2^	0.0245	0.0253	0.0101	0.0103

Note: *, *** indicate significance at the 10% and 1% levels, respectively.

**Table 4:** Results of heterogeneity analysis.

Variable	Self-Rated Health				Mental Health			
	Primary and Below	Secondary	University	Graduate	Primary and Below	Secondary	University	Graduate
Mobile Internet Usage	0.097 (0.072)	0.152 *** (0.058)	0.316 * (0.174)	−1.086 (1.166)	−0.094 (0.077)	−0.107 * (0.062)	−0.041 (0.187)	1.095 (1.187)
Control Variables	Yes	Yes	Yes	Yes	Yes	Yes	Yes	Yes
N	1027	4541	2270	130	1023	4540	2269	130
Adj-R^2^	0.0166	0.0277	0.0178	0.0404	0.0048	0.0104	0.0106	0.0507

Note: *, *** indicate significance at the 10% and 1% levels, respectively.

**Table 5:** Sample matching quality balance test.

Variable	Before After	Mean		Bias (%)	Reduce Bias (%)	T-Test	
		Treated	Control			t	*p* > |t|
Gender	B	0.339	0.291	10.2	76.7	2.70	0.007
	A	0.338	0.349	−2.4		−1.39	0.163
Age	B	29.90	36.035	−67.6	81.0	−20.24	0.000
	A	29.89	28.734	12.8		8.41	0.000
Marital Status	B	1.703	1.911	−37.3	95.7	−9.61	0.000
	A	1.705	1.696	1.6		0.85	0.393
Education level	B	2.254	1.599	106.2	88.2	27.54	0.000
	A	2.248	2.171	12.5		7.56	0.709
Medical Insurance	B	0.867	0.876	−2.6	92.4	−0.70	0.486
	A	0.867	0.866	0.2		0.12	0.907
Smoking status	B	0.070	0.058	5.1	−2.1	1.31	0.189
	A	0.069	0.057	5.2		3.11	0.002
Drinking situation	B	0.044	0.060	−7.3	92.3	−2.07	0.038
	A	0.044	0.043	0.6		0.36	0.716
Frequency of physical exercise	B	1.692	1.378	31.3	90.4	7.94	0.000
	A	1.686	1.656	3.0		1.64	0.101

Note: Treated means treated group; control means control group.

**Table 6:** Propensity score matching estimation results.

	Self-Rated Health				Mental Health			
	Treated	Control	ATT	SE	Treated	Control	ATT	SE
Before matching	2.581	2.639	0.057	0.038	1.558	1.646	−0.087	0.024
After matching								
K-nearest-neighbor matching	2.583	2.399	0.183	0.087	1.559	1.622	−0.063	0.053
K Nearby Caliper Matching	2.583	2.389	0.194	0.085	1.559	1.631	−0.072	0.052
Radius neighbor matching	2.583	2.383	0.199	0.072	1.559	1.626	−0.067	0.044
kernel matching	2.583	2.356	0.227	0.065	1.559	1.637	−0.078	0.040

Note: K-nearest-neighbor matching uses “one-to-four” matching, K-nearest-neighbor caliper matching and radius-neighbor matching have a radius of 0.01, and the default values of kernel function and bandwidth are used in kernel matching.

## Data Availability

The data of CFPS2020 are publicly available at http://www.isss.pku.edu.cn/cfps/ accessed on 30 December 2021.

## References

[1] Gu H., Wu D. (2021). The basic connotation and strategic conception of the high-quality development of the basic medical security system during the “14th Five-Year Plan” period. Manag. World.

[2] Yang L., Song L. (2022). A Decomposition Study of the Differences in Healthy Life Expectancy of the Chinese Elderly Population. Popul. Econ..

[3] Zhong R., Duan L. (2021). Xi Jinping’s Important Discourse on Healthy China and Its Significance. Theor. Vis..

[4] Report of the 19th Congress of the Communist Party of China. http://www.gov.cn/zhuanti/2017-10/27/content_5234876.htm.

[5] Highlights of the Communiqué of the Fifth Plenary Session of the Nineteenth Central Committee. http://cpc.people.com.cn/n1/2020/1029/c164113-31911575.html.

[6] Li L., Ding H., Li Z. (2022). Does Internet Use Impact the Health Status of Middle-Aged and Older Populations? Evidence from China Health and Retirement Longitudinal Study (CHARLS). Int. J. Environ. Res. Public Health.

[7] National Bureau of Statistics (2020). Statistical Communiqué of the People’s Republic of China on the 2020 National Economic and Social Development.

[8] von Rosen A.J., von Rosen F.T., Tinnemann P., Müller-Riemenschneider F. (2017). Sexual health and the internet: Cross-sectional study of online preferences among adolescents. J. Med. Internet Res..

[9] Duplaga M. (2021). The association between Internet use and health-related outcomes in older adults and the elderly: A cross-sectional study. BMC Med. Inform. Decis. Mak..

[10] Rapport D.J., Howard J., Lannigan R., McCauley W. (2003). Linking health and ecology in the medical curriculum. Environ. Int..

[11] Yang K., He H. (2020). Impact of Internet Use on the Health of the Population—A Study Based on Data from the 2016 China Labor Force Dynamics Survey. Nankai Econ. Res..

[12] Zhao J., Liu Z. (2020). Impact of Internet use on the health of older adults. China Pop Sci..

[13] Li L., Ding H. (2022). The Relationship between Internet Use and Population Health: A Cross-Sectional Survey in China. Int. J. Environ. Res. Public Health.

[14] Yang N., Gu H. (2020). Internet Use, Informal Social Support, and Farmers’ Health-Based on Chinese Household Tracking Survey Data. Rural Econ..

[15] Xu Y., Lai D. (2021). A Study of Internet Use, Risk Perceptions, and the Health of Urban Residents. J. Party Sch. Centr. Com. Party China (Nat. Sch. Adm.).

[16] Niu G., Shi X., Tian Y., Sun X., Lei Y. (2021). Social networking site use and depression in older adults: The role of online social capital and loneliness. Chin. J. Clin. Psychol..

[17] Matusitz J., McCormick J. (2012). Sedentarism: The effects of Internet use on human obesity in the United States. Soc. Work Public Health.

[18] Hökby S., Hadlaczky G., Westerlund J., Wasserman D., Balazs J., Germanavicius A., Machín N., Meszaros G., Sarchiapone M., Värnik A. (2016). Are mental health effects of internet use attributable to the web-based content or perceived consequences of usage? A longitudinal study of European adolescents. JMIR Ment. Health.

[19] Choi M., Park S., Cha S. (2017). Relationships of mental health and internet use in Korean adolescents. Arch. Psychiatr. Nurs..

[20] Ning K., Zhu Z., Xu Z. (2019). Internet, life time allocation and physical health of rural adolescents. Nankai Econ Res..

[21] Cheng X., Jiang Q. (2021). Social isolation and self-rated health of the elderly: The mediating role of aging attitudes. Popul. Dev..

[22] Zheng C., Wang X., Sun Q. (2021). Urban and rural medical insurance overall policy, residents’ health and research on health inequality. Nankai Econ. Res..

[23] Wang Y. (2020). A study on the impact of smartphone use on the subjective health of the elderly: Based on the data of the 2016 China Social Tracking Survey of the Elderly (CLASS). Popul. Dev..

[24] Wang S., Nie Y., Sutherland J.M., Wang L. (2021). Pattern discovery of health curves using an ordered probit model with Bayesian smoothing and functional principal component analysis. Stat. Methods Med. Res..

[25] Azra Batool S., Ahmed H.K., Qureshi S.N. (2018). Impact of demographic variables on women’s economic empowerment: An ordered probit model. J. Women Aging.

[26] Benedetto U., Head S.J., Angelini G.D., Blackstone E.H. (2018). Statistical primer: Propensity score matching and its alternatives. Eur. J. Cardio-Thorac. Surg..

[27] Austin P.C., Jembere N., Chiu M. (2018). Propensity score matching and complex surveys. Stat. Methods Med. Res..

[28] Caliendo M., Kopeinig S. (2008). Some practical guidance for the implementation of propensity score matching. J. Econ. Surv..

[29] Dehejia R.H., Wahba S. (2002). Propensity score-matching methods for nonexperimental causal studies. Rev. Econ. Stat..

[30] Lu J., Wang B. (2020). Research on the Mechanism of Residents’ Internet Use on Their Self-evaluation Health Impact—Based on the 2016 Chinese Family Panel Studies Data. J. Sun Yat-Sen Univ. (Soc. Sci. Ed.).

[31] Li L., Ding H. (2022). Internet Use, Leisure Time and Physical Exercise of Rural Residents—An Empirical Analysis Based on 2018 CFPS Data. Lanzhou Acad. J..

[32] Zhangm X., Yang T., Wang C., Wan G. (2020). Digital Finance Development and Resident Consumption Growth: Theory and Practice in China. Manag. World.

[33] Royant-Parola S., Londe V., Tréhout S., Hartley S. (2017). The use of social media modifies teenagers’ sleep-related behavior. L’encephale.

[34] Liu J., Guo C. (2021). Effects of mobile Internet application (APP) use on physical and mental health of older adults: The use of WeChat, WeChat Friend Circle and mobile payment as examples. Pop Dev..

[35] Mylona I., Deres E.S., Dere G.-D.S., Tsinopoulos I., Glynatsis M. (2020). The impact of internet and videogaming addiction on adolescent vision: A review of the literature. Front. Public Health.

[36] Liu Y., Ni X., Niu G. (2021). Perceived Stress and Short-Form Video Application Addiction: A Moderated Mediation Model. Front. Psychol..

[37] Sun X., Duan C., Niu G., Tian Y., Zhang Y. (2021). Mindfulness buffers the influence of stress on cue-induced craving for Internet among Chinese colleges with problematic Internet use. J. Behav. Addict..

[38] Xie L., Yang H.-L., Lin X.-Y., Ti S.-M., Wu Y.-Y., Zhang S., Zhang S.-Q., Zhou W.-L. (2021). Does the Internet Use Improve the Mental Health of Chinese Older Adults?. Front. Public Health.

[39] Pantic I. (2014). Online social networking and mental health. Cyberpsychology Behav. Soc. Netw..

[40] Chambers D., Cairns K., Ivancic L. (2018). Young people, the internet and mental health. Ir. J. Psychol. Med..

[41] Wen Y., Ding Y. (2022). Analysis of the influence of Internet use on life well-being of middle-aged and elderly people. Popul. Health.

[42] Chen X., Yang H. (2021). The influence of the Internet on the subjective well-being of rural residents and its mechanism analysis. J. Agric. For. Econ. Manag..

[43] Chandra S., Prasad N.R., Lindgren P., Prasad R. (2022). C5: A Step Towards Smart World with Enhanced Holistic Wellbeing. Wirel. Pers. Commun..

[44] Zhang H., Wang H., Yan H., Wang X. (2021). Impact of Internet Use on Mental Health among Elderly Individuals: A Difference-in-Differences Study Based on 2016–2018 CFPS Data. Int. J. Environ. Res. Public Health.

[45] Yang H.-L., Wu Y.-Y., Lin X.-Y., Xie L., Zhang S., Zhang S.-Q., Ti S.-M., Zheng X.-D. (2021). Internet use, life satisfaction, and subjective well-being among the elderly: Evidence from 2017 China general social survey. Front. Public Health.

[46] Wang L. (2018). A study on the mechanism of the impact of Internet use on physical and mental health of the elderly—An empirical analysis based on CGSS (2013) data. Mod. Econ. Discuss..

[47] Ran X., Hu H. (2022). Rural-urban disparities, digital divides and health inequalities in older age. Demogr. J..

[48] Yang J., Liu Y. (2022). Longevity Dividend in the Digital Age: Feasible Capabilities and Endogenous Motivation in the Digital Life of the Elderly. Adm. Reform.

[49] Sun X., Zhang Y., Niu G., Tian Y., Xu L., Duan C. (2021). Ostracism and Problematic Smartphone Use: The Mediating Effect of Social Self-Efficacy and Moderating Effect of Rejection Sensitivity. Int. J. Ment. Health Addict..

